# GladiaTOX: GLobal Assessment of Dose-IndicAtor in TOXicology

**DOI:** 10.1093/bioinformatics/btz187

**Published:** 2019-03-14

**Authors:** Vincenzo Belcastro, Stephane Cano, Diego Marescotti, Stefano Acali, Carine Poussin, Ignacio Gonzalez-Suarez, Florian Martin, Filipe Bonjour, Nikolai V Ivanov, Manuel C Peitsch, Julia Hoeng

**Affiliations:** Philip Morris Products S.A, PMI R&D, Neuchâtel CH, Switzerland

## Abstract

**Summary:**

GladiaTOX R package is an open-source, flexible solution to high-content screening data processing and reporting in biomedical research. GladiaTOX takes advantage of the ‘tcpl’ core functionalities and provides a number of extensions: it provides a web-service solution to fetch raw data; it computes severity scores and exports ToxPi formatted files; furthermore it contains a suite of functionalities to generate PDF reports for quality control and data processing.

**Availability and implementation:**

GladiaTOX R package (bioconductor). Also available via: git clone https://github.com/philipmorrisintl/GladiaTOX.git.

**Supplementary information:**

Supplementary data are available at *Bioinformatics* online.

## 1 Introduction

Drug discovery has historically relied on the massive screening of compound libraries with *in vitro* cell-based target assays. The challenges of performing larger screening campaigns while keeping time and costs limited have led the pharmaceutical industry to develop *in vitro* high-content screening (HCS) technologies. These techniques, generating thousands of data points per day obtained by performing several compound dose-response investigations in multiple assays, require standardized and robust data analysis procedures that can enable rapid decision making.

Quality control (QC), processing and reporting are the typical steps applied to experimental HCS data. During QC, all images’ control parameters are inspected against standard acceptance criteria. Processing includes data normalization, dose-response fitting and evaluation of standard parameters, such as the minimal effective concentration (MEC). Analysis results are then shared via formatted reports. Deficient analysis pipelines can lead to long, untraceable procedures. Existing software for HCS data analysis is limited due to the lack of formatted reporting (File *et al.*, 2017; Fourches *et al.*, 2014) and limited to specific platforms (Di Veroli *et al.*, 2015). There also exist commercial packages which would require licensing fees (e.g. OriginLab, GraphPad and Screener). GladiaTOX, provides transparent data processing opportunities, it is configurable to different assay set up and to any plate design.

**Fig. 1.**
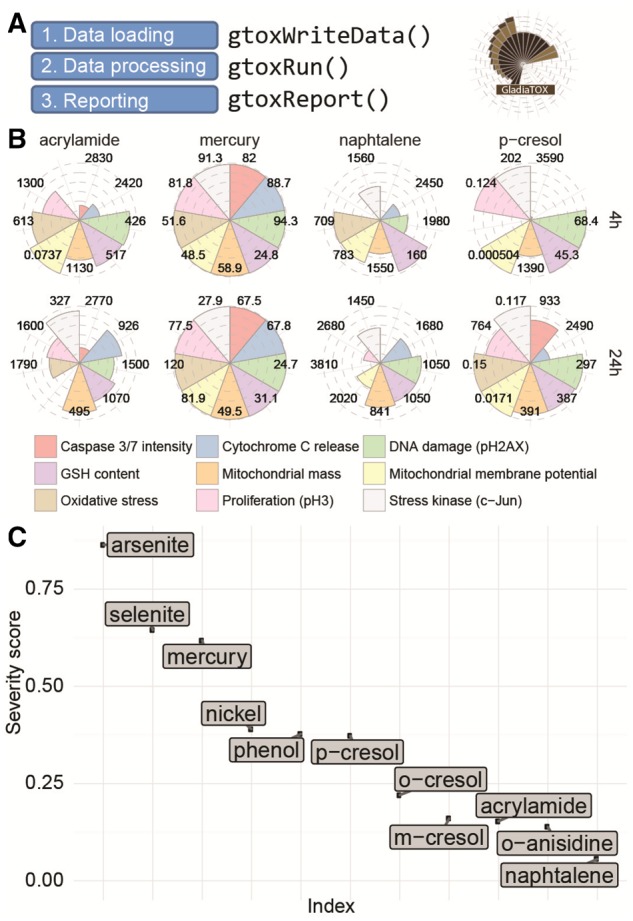
Processing workflow and reporting plots. (**A**) Simplified analysis workflow with package function calls. (**B**) MEC plot. A circular plot is shown for each chemical and endpoint. Numbers indicate MEC values (μM). Filled slices indicate higher toxicity (low MEC). MECs for only four chemicals are reported, at 4 and 24 h. (**C**) Severity score plot. On the *y*-axis, chemicals are sorted according to severity score values. Higher scores are associated with higher toxicity

GladiaTOX is an open-source solution for HCS data processing and reporting (Fig. 1) that expands the tcpl package from the ToxCast pipeline (Filer *et al.*, 2017). In addition to tcpl’s functionalities (multiple dose-response fitting and best fit selection), GladiaTOX (i) fetches raw image quantifications via a web service, allowing proprietary systems to be integrated; (ii) computes MEC using historical data; (iii) exports results formatted for the ToxPi graphical user interface (Marvel *et al.*, 2018; Reif *et al.*, 2010); (iv) aggregates single-endpoint MECs into severity scores and (v) implements a suite of functionalities for QC and processing reports in a structured PDF format. The effects of 11 chemicals, investigated using an image-based high-throughput application (Marescotti *et al.*, 2016) are reported as a use case (Supplementary Materials). Overall, metals showed low MEC values (high toxicity) for most endpoints tested (including cytotoxicity, DNA damage and oxidative stress). The severity scores ranked metals as more toxic than phenols and assigned low severity to acrylamide and naphthalene.

## 2 Results

In tcpl (Filer *et al.*, 2017) all related experimental data are grouped under the same study. GladiaTOX database adds granularity by defining study phases as an additional level to group related experimental data. Each study phase is associated to a list of endpoints, chemicals tested, exposures, and concentrations. There are two ways to load raw data into the database: from Excel files, or directly from the instrument database.

### 2.1 Data normalization and processing

Data normalization is one step in HCS data processing. GladiaTOX implements a multitude of normalization functions. To see the full list, type: gtoxMthdList(lvl = 3). Different treatments can share the same vehicle, and GladiaTOX allows different vehicles to be used in the same plate by defining vehicle IDs.

Three functions are used to model dose-response series (see Supplementary materials and Supplementary Fig. S1A). Dose-response signals are validated against random signals to account for noise in the data (gray band in Supplementary Fig. S1B). The tcpl package assumes that zero response should be observed for the lowest two concentrations of a series. Based on this assumption, cutoff values are computed. The assumption is not necessarily true, especially for small plates, where a limited number of concentrations can be tested. GladiaTOX overcomes this constraint by allowing the user to define noise-band margins from vehicle responses. Signal noise is important, as multiple statistics use this cutoff (e.g. MEC; Supplementary Fig. S1B). Cutoff values can also be computed from historical data and per endpoint.

### 2.2 Reporting

Reporting is a software module exclusive to GladiaTOX that offers QC and processing reports. Both types are exported to PDF with the original figures for presentations or publications.

The QC report sections are organized per assay, it includes summary tables with number of samples tested and a list of processing methods applied per endpoint. Raw data heatmaps and positive control dose-response plots are two QC plots useful for diagnostics (Supplementary Fig. S2A) and show the heatmap of image quantification values before normalization. Supplementary Figure S2B shows the response of the positive control for the apoptosis/necrosis assay. The plate in the example has passed QC, because at least one concentration has response values beyond the noise band.

The data processing report includes the study overview, stimulus summary and assay summary. The study overview section contains a description of the study, a table listing the assays and endpoints, and a stimulus overview table listing the number of samples tested for each chemical in the study (Supplementary Table S1). The stimulus summary section includes details on the chemicals tested. The summary table (Supplementary Fig. S3) reports, for each endpoint, the model that best fits the data and a set of statistics including MEC (Supplementary Fig. S1). For each endpoint or time point, dose-response plots show the model that best fits the data for each replicated plate. The assay summary section contains an overview table (Supplementary Fig. S4) with the number of compounds tested for each endpoint and the number of active compounds (at least one response value outside the grey band). Additional information includes the list of methods applied to transform and normalize the data.

Figure 1B and C displays additional reporting plots that can be generated in GladiaTOX. The plot in Figure 1B was generated with the function glPlotPie(). MEC values are shown for each chemical/time point. Lower MECs are associated with higher toxicity (see mercury). The MEC average impact is then summarized as a severity score (Fig. 1C) that represents the overall impact of chemicals across multiple endpoints (arsenite has the highest score).

## 3 Conclusions

The GladiaTOX package, with its suite of functionalities, represents an all-in-one, open-source, flexible solution to store, process and report HCS data in biomedical research.

## Supplementary Material

btz187_Supplementary_Data
