# Supplementary material for: GladiaTOX: GLobal Assessment of Dose-IndicAtor in TOXicology
Source: Bioinformatics. 2019 Mar 14;35(20):4190–2. doi: 10.1093/bioinformatics/btz187 (PMC6792086; doi:10.1093/bioinformatics/btz187)
Supplement: btz187_Supplementary_Data [file btz187_supplementary_data.docx]

**Supplementary materials**

GladiaTOX: GLobal Assessment of Dose-IndicAtor in Toxicology

Vincenzo Belcastro, Stephane Cano, Diego Marescotti, Stefano Acali, Carine Poussin, Ignacio Gonzalez-Suarez, Florian Martin, Filipe Bonjour, Nikolai V. Ivanov, Manuel C. Peitsch, and Julia Hoeng

1 PMI R&D, Philip Morris Products S.A., Quai Jeanrenaud 5, CH-2000 Neuchâtel, Switzerland

Table of Contents

[1 Cell cultures 2](#_Toc533063943)

[2 Chemicals 2](#_Toc533063944)

[3 Assay/endpoint table 4](#_Toc533063945)

[4 HCS data processing 4](#_Toc533063946)

[5 Reporting and statistics 5](#_Toc533063947)

[6 Supplemetary figures and tables 5](#_Toc533063948)

[7 Data availability 8](#_Toc533063949)

# Cell cultures

Normal human bronchial epithelial (NHBE) cells were purchased from Lonza (Catalog no. CC-2540, Lonza, Cologne, Germany). The donor was a 60-year-old Caucasian male with no history of smoking. The cells were maintained in a humidified incubator at 37°C and 5% CO_2_ and cultured in bronchial epithelial cell medium (Bullet Kit CC 3170, Lonza) according to the vendor’s recommendations.

Human coronary artery endothelial cells (HCAEC) were purchased from Vitaris (Catalog no. 12221-PRO, Baar, Switzerland). The donor was a 43-year-old Caucasian healthy female with no history of smoking. The cells were maintained in a humidified incubator at 37°C and 5% CO_2_ and cultured in MV2 medium (22022-PRO, Vitaris) according to the vendor’s recommendations.

# Chemicals

| **Toxicant** | **Vehicle** | **Doses (µM)** | | | | | |
| --- | --- | --- | --- | --- | --- | --- | --- |
|  |  | **1** | **2** | **3** | **4** | **5** | **6** |
| ***Acid derivatives*** |  |  |  |  |  |  |  |
| Acrylamide | *Water* | 10000 | 5000 | 2500 | 1250 | 1.2E-01 | 4.0E-02 |
| ***Aromatic amines*** |  |  |  |  |  |  |  |
| O-Anisidine | *Ethanol* | 10000 | 5000 | 1000 | 200 | 7.0E-05 | 6.0E-07 |
| ***Phenols*** |  |  |  |  |  |  |  |
| Phenol | *Ethanol* | 5000 | 2000 | 1000 | 500 | 2.4E-01 | 1.6E-02 |
| M-Cresol | *Ethanol* | 5000 | 2000 | 1000 | 500 | 5.0E-02 | 3.0E-04 |
| O-Cresol | *Ethanol* | 5000 | 2000 | 1000 | 500 | 7.5E-04 | 9.0E-07 |
| P-Cresol | *Ethanol* | 5000 | 2000 | 1000 | 500 | 1.5E-01 | 8.0E-04 |
| ***PAHs*** |  |  |  |  |  |  |  |
| Naphthalene | *Ethanol** | 5000 | 2500 | 1250 | 625 | 2.0E-02 | 8.0E-05 |
| ***Metals/ elements*** |  |  |  |  |  |  |  |
| Mercury | *Ethanol* | 160 | 120 | 80 | 40 | 4.0E-05 | 8.0E-06 |
| Nickel | *Water* | 2000 | 1000 | 200 | 100 | 3.4E-05 | 1.4E-05 |
| Arsenite | *Water* | 50 | 25 | 12.5 | 6.25 | 1.7E-04 | 3.0E-05 |
| Selenite | *Water* | 500 | 250 | 200 | 125 | 3.5E-04 | 2.0E-05 |

**Supplementary Table 1.** List of chemicals and concentrations used in NHBE cells. (*: vehicle was 1.75% EtOH + 0.25% dimethyl sulfoxide [DMSO]).

| **Toxicant** | **Vehicle** | **Doses (µM)** | | | | | |
| --- | --- | --- | --- | --- | --- | --- | --- |
|  |  | **1** | **2** | **3** | **4** | **5** | **6** |
| ***Acid derivatives*** |  |  |  |  |  |  |  |
| Acrylamide | *Water* | 1250 | 625 | 312.5 | 156.25 | 1.2E-01 | 4.0E-02 |
| ***Aromatic amines*** |  |  |  |  |  |  |  |
| O-Anisidine | *Ethanol* | 5000 | 1250 | 625 | 312.5 | 7.0E-05 | 6.0E-07 |
| ***Phenols*** |  |  |  |  |  |  |  |
| Phenol | *Ethanol* | 5000 | 2500 | 625 | 312.5 | 2.4E-01 | 1.6E-02 |
| M-Cresol | *Ethanol* | 2000 | 500 | 100 | 50 | 5.0E-02 | 3.0E-04 |
| O-Cresol | *Ethanol* | 2000 | 500 | 100 | 50 | 7.0E-02 | 6.0E-04 |
| P-Cresol | *Ethanol* | 2000 | 500 | 100 | 50 | 1.5E-01 | 8.0E-04 |
| ***PAHs*** |  |  |  |  |  |  |  |
| Naphthalene | *Ethanol** | 1000 | 500 | 200 | 100 | 2.0E-02 | 8.0E-05 |
| ***Metals/ elements*** |  |  |  |  |  |  |  |
| Mercury | *Ethanol* | 80 | 16 | 3.2 | 0.64 | 4.0E-05 | 8.0E-06 |
| Nickel | *Water* | 1250 | 625 | 312.5 | 156.25 | 4.0E-05 | 1.3E-05 |
| Arsenite | *Water* | 10 | 2 | 1 | 0.4 | 1.7E-04 | 3.0E-05 |
| Selenite | *Water* | 800 | 400 | 200 | 100 | 4.0E-05 | 2.0E-05 |

**Supplementary Table 2.** List of chemicals used in HCAEC cells. (*: vehicle was 1.75% EtOH + 0.25% DMSO).

The list of environmental toxicants used and the doses tested on NHBE and HCAEC in the study are detailed in Supplementary Table 1 and Supplementary Table 2, respectively. All chemicals were purchased from Sigma-Aldrich (St. Louis, MO, USA) at the highest available purity grade. The list include four metals (mercury, nickel, arsenite, and selenite), four phenols (phenol, m-cresol, o-cresol, and p-cresol), acrylamide, o-Anisidine, and naphthalene.

# Assay/endpoint table

Supplementary Table 3 shows the list of assays measured in the study.

| **Assay** | **Endpoint** | **Positive control** | **Analysis direction** |
| --- | --- | --- | --- |
| **Cytotoxicity** | Mitochondrial mass | CCCP | Up/down |
|  | Mitochondrial membrane potential |  | Up/down |
|  | Cytochrome C release |  | Up |
| **DNA damage & stress kinase** | Phospho-H2AX | CCCP | Up |
|  | Phospho-cJun |  | Up |
| **Oxidative stress** | Reactive oxygen species | Ethacrynic acid | Up/down |
|  | Glutathione |  | Up/down |
| **Apoptosis & necrosis** | Caspase 3/7 | CCCP | Up |
|  | Cell membrane permeability |  | Up |

**Supplementary Table 3.** HCS assays with relative endpoints and reference compounds.

# HCS data processing

HCS is a set of analytical methods in which multiple fluorescence readouts are measured simultaneously (Marescotti, et al., 2016). The quantified fluorescence images were stored in the GladiaTOX database. As QC (Bray and Carpenter, 2004), positive controls were first analysed to ensure that staining procedures had been performed correctly. Data not passing QC were masked. Raw data passing QC were normalised to vehicles using the following equation:

$$N\left( i \right)=log2\left( \frac{i}{Veh} \right)$$

where i is the measured raw signal value of a well, and Veh is the median of the measured signal values for the vehicle wells on a plate. The full list of normalization functions can be explored with the command:

gtoxMthdLoad(lvl=3).

Dose-response normalized data were modelled using three fitting functions: Constant, Hill, and Gain-loss (Supplementary Figure 1A). Fitting directions for each endpoint are defined in Supplementary Table 1 (up, positive; down, negative). The best-fitting model among the three (minimizing the Akaike information criterion) was retained.

# Reporting and statistics

The model functions that best fit the data were used to compute the MEC (Supplementary Figure 1B). MECs are identified as the inter-section of the fitted curve with the noise band (grey band in figure). The noise band was computed as three times the baseline median absolute deviation of vehicle responses. It is good practice to pull in the same-plate chemicals sharing one vehicle to maximize the number of points used to compute the noise band. MEC values were not available in the case of constant fit (modelled activity not intersecting the noise band).


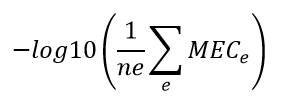
Severity scores are toxicological indicator values. For each chemical, the severity score is computed with the following formula:

where ne is the number of tested endpoints, e is a generic end-point, and MECe is the MEC for endpoint e. The severity score expresses the average impact of chemicals across the list of end-points tested. Larger values correspond to higher chemical toxicity. Severity scores are scaled in the interval [0, 1].

# Supplemetary figures and tables

**
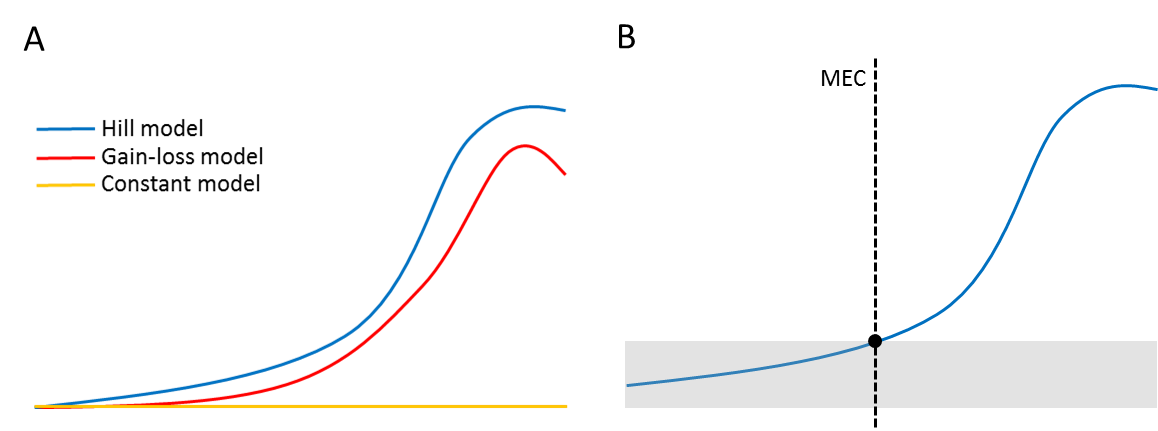
**

**Supplementary Figure 1.** Example of fitting models and minimal effective concentration (MEC). (A) The three models utilized by the GladiaTOX package. (B) Identification of the MEC value. The MEC (black dot) value is the concentration (*x*-axis) at which the fitted model (blue line) intersects the cutoff (grey band, median absolute deviation of vehicle responses). This concentration is also referred to as activity concentration at cutoff (ACC).


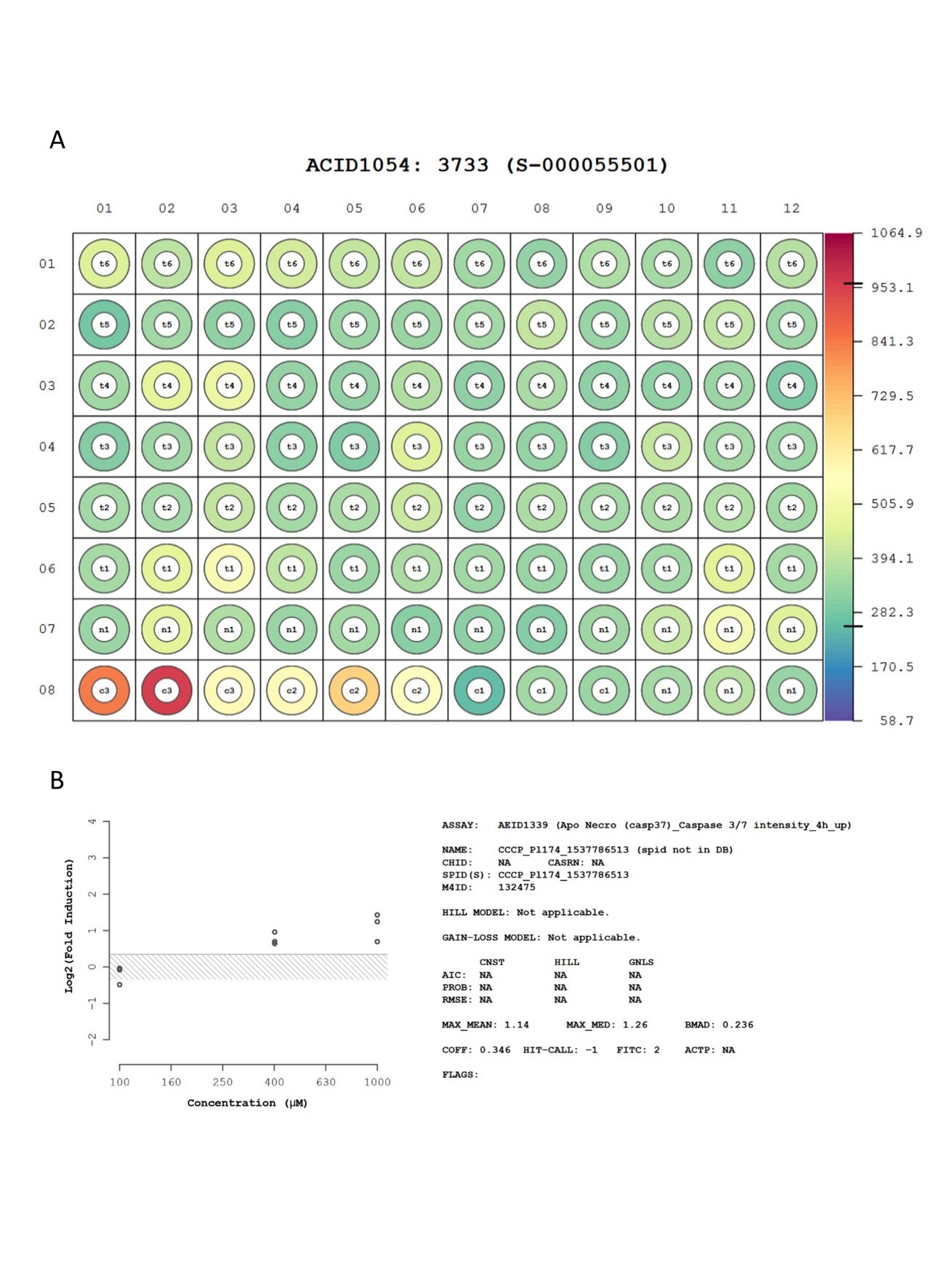


**Supplementary Figure 2.** Example of quality control output. (A) Image of raw data heatmap of a plate. Plate title contains the assay component identification number (1054), the plate identification number (3733), and the plate name (S-000055501). Plate dimension is 8x12. Letters and numbers in each well indicate the well type (t: treatment, c: positive control, n: neutral control or vehicle), and the concentration index (from low to high), respectively. Row 8 displays the values of the assay positive control (CCCP in the example). (B) Positive control dose-response plot is shown on the right. Here plotted values are already normalized against the vehicle (log_2_ fold change). The grey band indicates vehicle variability (see Methods in the main text). The right panel reports additional information, such as endpoint name (ASSAY) and noise band margin (COFF).


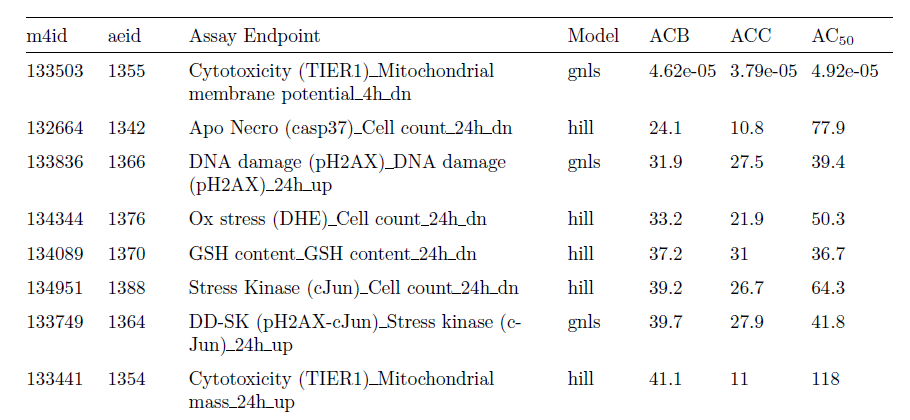


**Supplementary Figure 3.** Compound summary table. For each endpoint, the table shows the model that best fits the data and a set of statistics, including the activity concentration at baseline, cutoff, and at 50% response (ACB, ACC, and AC_50_, respectively).


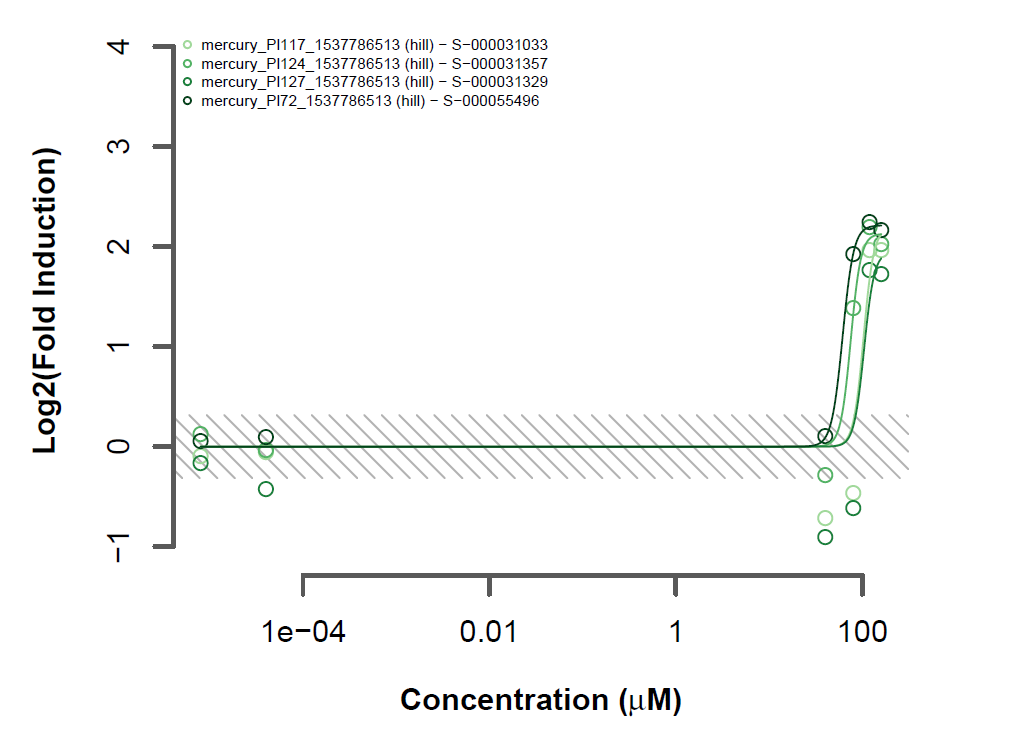


**Supplementary Figure 4.** Example concentration-response plots. The figure shows, for each of the four plates listed in the legend, the model that best fits the data. Each green circle represents the response value measured on a plate. In the example, responses are computed as the log_2_ fold change of treatment over vehicle raw values. The grey band reflects vehicle variability (three times the baseline median absolute deviation of vehicle responses).


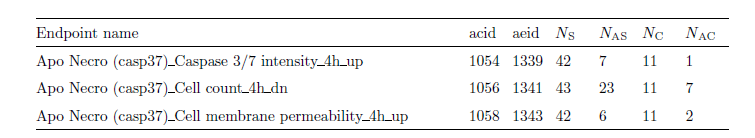


**Supplementary Figure 5.** Endpoint summary table. In the example, the apoptosis/necrosis assay is displayed. For each assay endpoint, the table shows internal database ids (acid and aeid); *N*_S_ is the total number of samples tested across all assay endpoints, *N*_AS_ is the number of active samples, *N*_C_ is the number of compounds tested on the endpoint, and *N*_AC_ is the number of active compounds.

| Compound | *N*_S_ | *N*_AS_ | *N*_E_ | *N*_AE_ |
| --- | --- | --- | --- | --- |
| Acrylamide | 166 | 65 | 44 | 37 |
| Arsenite | 120 | 44 | 52 | 28 |
| M-Cresol | 164 | 84 | 52 | 39 |
| Mercury | 164 | 117 | 52 | 41 |
| Naphthalene | 208 | 79 | 52 | 36 |
| Nickel | 220 | 57 | 44 | 23 |
| O-Anisidine | 182 | 69 | 46 | 27 |
| O-Cresol | 164 | 88 | 52 | 37 |
| P-Cresol | 120 | 61 | 52 | 35 |
| Phenol | 132 | 46 | 44 | 26 |
| Selenite | 164 | 88 | 52 | 37 |

**Supplementary Table 4.** Overview of tested compounds. *N*_S_ is the total number of samples across all endpoints, *N*_AS_ is the number of active samples, *N*_E_ is the number of unique endpoints tested, and *N*_AE_ is the number of unique endpoints activated by the compound.

# Data availability

HCS data is available at <https://www.intervals.science/>
